# Efficacy of outdoor interventions for myopia in children and adolescents: a systematic review and meta-analysis of randomized controlled trials

**DOI:** 10.3389/fpubh.2024.1452567

**Published:** 2024-08-13

**Authors:** Zhengyang Mei, Yuanzhuo Zhang, Wenfeng Jiang, Chifong Lam, Shulai Luo, Chenyi Cai, Shi Luo

**Affiliations:** ^1^School of Physical Education, Southwest University, Chongqing, China; ^2^Key Laboratory of Cognition and Personality, Faculty of Psychology, Ministry of Education, Southwest University, Chongqing, China

**Keywords:** children, adolescents, outdoor interventions, myopia, meta-analysis

## Abstract

**Objectives:**

The objective of this systematic review and meta-analysis was to evaluate the overall efficacy of outdoor interventions for myopia in children and adolescents, and to provide evidence for the prevention and control of myopia.

**Methods:**

Randomized controlled trials of outdoor interventions for myopia in children and adolescents were identified using electronic databases and manual searches. The Revised Cochrane risk-of-bias tool for randomized trials (RoB 2) was used to assess risk of bias in randomized controlled trials. A mean difference (MD) and a risk ratio (RR) with a 95% confidence interval (CI) were used to combine effect sizes. A sensitivity analysis was performed for each outcome using a stepwise elimination method to assess whether the pooled results were significantly affected by individual studies.

**Results:**

The analysis included seven randomized controlled trials involving a total of 9,437 subjects. The meta-analysis showed marked and statistically significant improvements in spherical equivalent refraction (MD = 0.19; 95% CI 0.14 to 0.25; *p* < 0.01), axial length (MD = −0.09; 95% CI −0.13 to −0.05; *p* < 0.01), and myopia incidence (RR = 0.84; 95% CI 0.78 to 0.91; *p* < 0.01) following outdoor interventions.

**Conclusion:**

Outdoor interventions effectively contributed to the prevention and control of myopia in children and adolescents, positively impacting spherical equivalent refraction, axial length, and myopia incidence. Outdoor interventions were characterized by low risk and high therapeutic benefits and could serve as alternative or adjuvant approaches to medication for the treatment of myopia. Considering the advantages in terms of safety and efficacy, outdoor interventions may be considered as a preferred intervention for the treatment of myopia in children and adolescents, while susceptibility to diseases associated with sunlight, particularly UV exposure, must be taken into account.

**Systematic review registration:**

https://www.crd.york.ac.uk/prospero/, Identifier CRD42024538695.

## 1 Introduction

Myopia, as one of the most common public health problem in the world, is a major eye disease leading to visual impairment in children and adolescents (1, 2). In recent years, the way children and adolescents access information has been altered dramatically with the changes in the global economy and social environment, as well as the widespread popularity of smart electronic products and the emergence of online we-media. Significant alterations in the learning pathways, lifestyles and sleeping habits of children and adolescents have had a profound impact on myopia, and the situation for myopia prevention and control has become increasingly challenging (3, 4). The current global prevalence of myopia is estimated to be in excess of 28.3%, with projections indicating that by 2050, the coverage will reach 49.8%, while the prevalence of high myopia will also reach 10% (5, 6). Meanwhile, a substantial body of evidence has indicated that myopia is particularly severe in certain demographic groups, especially among children and adolescents, with an overall myopia incidence exceeding 50% (7–9). It should be emphasized that high myopia increases the risk of pathologic ocular changes, including cataract, glaucoma, retinal detachment, and myopic macular degeneration, which may lead to irreversible vision loss (10, 11).

For children and adolescents, the heavy educational burden and the prevalence of smart electronic products have led to a sharp increase in the need for long-term short-distance use of eyes (e.g., reading, writing, and using electronic devices), thus resulting in a significant increase in the probability of myopia in this group, with a notable increase in the number of cases diagnosed at an early age and with a high degree of severity (12). Several countermeasures have been developed to help children and adolescents effectively prevent and control myopic progression, including Atropine (13), Pirenzepine (14), Orthokeratology (15), Spectacle lenses (16), and Contact lenses (17). However, these medications have certain drawbacks, including the potential for developing drug resistance with prolonged use, the risk of rebound upon discontinuation, and increased susceptibility to keratitis associated with long-term wearing of contact lenses (18, 19). In this context, outdoor interventions may help to address these limitations. As a self-directed health behavior, outdoor interventions (including engaging in outdoor activities and increasing time spent outdoors) have the advantages of being highly participatory and inexpensive, which are difficult to replace with drugs and lenses. While previous evidence suggested outdoor interventions effectively reduce the incidence and progression of myopia in children and adolescents, conflicting findings exist (20–24). Some studies indicated no direct association between outdoor interventions and myopia in this age group (25–27). Therefore, there is no unified consensus among experts on whether or not the progression of myopia in children and adolescents can be effectively prevented and controlled.

Myopia is generally quantified as spherical equivalent refraction (SER), which is commonly defined as the SER of ≤ −0.5 dioptres (D) or less after cycloplegic refraction (1, 28). In addition, axial length (AL) is one of the most important physiological indicators in the progression of myopia, and its change is closely related to refractive status, with longer AL implying more severe myopia (29–32). Therefore, control of AL of the eye during development is crucial for achieving normal vision, and therefore is a primary site for prevention (1). However, published randomized controlled trials (RCTs) of outdoor interventions to prevent and control myopia in children and adolescents provide inconsistent evidence, resulting in different effect sizes. For children and adolescents with an increasing myopia incidence, improving myopia through outdoor interventions rather than medication contributes to their physical and mental health development and quality of life (33, 34). The objective of this systematic review and meta-analysis was to evaluate the overall efficacy of outdoor interventions for myopia in children and adolescents, and to provide evidence for the prevention and control of myopia.

## 2 Methods

This systematic review and meta-analysis followed the Preferred Reporting Items for Systematic Reviews and Meta-Analyses (PRISMA 2020) and was registered in the International Prospective Register of Systematic Reviews (PROSPERO), under number CRD42024538695.

### 2.1 Search strategy

Based on medical subject headings and free-text terms, a search was conducted across six databases: PubMed, Embase, EBSCOhost, Scopus, Web of Science, and APA PsycINFO. Additionally, the Google database was manually searched for relevant studies. The search timeframe was from the inception of each database to April 2024, and the search strategy followed the PICOS principle: (P) population: children and adolescents (ages between 6 and 19 years); (I) intervention: outdoor interventions, including outdoor activities, time spent outdoors, outdoor exposure, etc.; (C) control: control group receiving only routine treatment or appropriate rehabilitation intervention; (O) outcome: any assessment of myopia; (S) study design: randomized controlled trials. The search strategy is presented in Table 1, per the PubMed database.

**Table 1:** PubMed search strategy.

#1	Myopi* [MeSH Terms] OR Refractive Errors [MeSH Terms]
#2	Myopi* [Title/Abstract] OR Myopia [Title/Abstract] OR Myopias [Title/Abstract] OR Short-sight [Title/Abstract] OR Short-sighted [Title/Abstract] OR Short-sightedness [Title/Abstract] OR Short sight [Title/Abstract] OR Short sighted [Title/Abstract] OR Short sightedness [Title/Abstract] OR Near-sight [Title/Abstract] OR Near-sighted [Title/Abstract] OR Near-sightedness [Title/Abstract] OR Near sight [Title/Abstract] OR Near sighted [Title/Abstract] OR Near sightedness [Title/Abstract] OR Refractive Errors [Title/Abstract] OR Refract* [Title/Abstract] OR Ametropi* [Title/Abstract]
#3	#1 OR #2
#4	Outdoor* [Title/Abstract] OR Outside [Title/Abstract] OR Physical activit* [Title/Abstract] OR Leisure activit* [Title/Abstract] OR Exercise* [Title/Abstract] OR Sport* [Title/Abstract]
#5	Adolescen* [Title/Abstract] OR Teen* [Title/Abstract] OR Youth* [Title/Abstract] OR Child* [Title/Abstract] OR Minor* [Title/Abstract] OR Pupil* [Title/Abstract] OR Pediatric* [Title/Abstract] OR Paediatric* [Title/Abstract]
#6	#3 AND #4 AND #5

### 2.2 Inclusion and exclusion criteria

The criteria for inclusion and exclusion of studies are presented in Table 2.

**Table 2:** Inclusion and exclusion criteria.

Category	Inclusion criteria	Exclusion criteria
Population	Children and adolescents groups (ages between 6 and 19 years)	Not children and adolescents
Intervention	Outdoor interventions, including outdoor activities, time spent outdoors, outdoor exposure, etc.	Not outdoor interventions
Control	Control group receiving only routine treatment or appropriate rehabilitation intervention	Inappropriate control conditions or control groups
Outcome	Any assessment for myopia, including spherical equivalent refraction, axial length, and myopia incidence	Studies that did not assess myopia
Study design	Randomized controlled trials	Non-randomized controlled trials, such as quasi-randomized controlled trials, study protocols, review, conference abstracts, comments, etc.

### 2.3 Studies selection and quality assessment

According to the predetermined inclusion and exclusion criteria, two independent researchers (ZYM and WFJ) used EndNote 20.6 bibliographic software for evidence selection. Duplicates were excluded when the references were imported into EndNote 20.6 and the remaining duplicates were manually removed. Two independent researchers screened and checked the references based on information such as the title, abstract, and full text. During the study selection process, any controversies were discussed and addressed by consulting the third author (SL).

The Revised Cochrane risk-of-bias tool for randomized trials (RoB 2) was used to assess the risk of bias in RCTs, in the following five aspects: (1) randomization process, (2) deviations from intended interventions, (3) missing outcome data, (4) measurement of the outcome, and (5) selection of the reported result. For each eligible study, the overall risk of bias was assessed as either low risk of bias, with some concerns, or high risk of bias. During the quality assessment process, any controversies were discussed and addressed by consulting the third author (SL).

### 2.4 Data extraction

Using a data extraction form that included relevant information, two independent researchers collected the following data from each included study: (1) basic information, including the first author, country, and year of publication, (2) participant characteristics, including mean (standard deviations) age, sample size, and percentage of boys, (3) intervention and control, and (4) myopia-related outcome measures.

### 2.5 Statistical analysis

All the outcomes assessed in this systematic review and meta-analysis included spherical equivalent refraction (SER), axial length (AL), and myopia incidence. For continuous variables, the mean difference (MD) with a 95% confidence interval (CI) was used to combine effect sizes as the measurement tools used in different RCTs were the same (35). For binary variables, the risk ratio (RR) was calculated with a 95% CI where the RR represents the ratio of the cumulative incidence of myopia between the intervention and control groups over the entire study period. For all meta-analyses, heterogeneity among studies was assessed using the Chi-square test based on *Q*-test and *I*^2^ statistics with a significance level of *p*-value < 0.10 (36). According to the recommendations of Cochrane’s handbook, when *p*-value <0.10 or *I*^2^ > 50%, there was a significant heterogeneity, and a random-effect model was used to merge the results. Otherwise, a fixed-effect model was used to merge the results when there was no significant heterogeneity (*p*-value >0.10 or *I*^2^ < 50%) (35). All meta-analyses of this study were performed using Stata 18.0 software.

Considering the number of studies included, publication bias was assessed by Egger’s test. The small-scale study effects were evaluated by calculating Egger’s regression intercepts, with *p*-value <0.05 as a threshold for statistical significance (37). The trim and fill method was used to assess the stability of the pooled results if there was publication bias (38). A sensitivity analysis was performed for each outcome using a stepwise elimination method to assess whether the pooled results were significantly affected by individual studies (35). The analysis showed that the pooled results remained stable and were not substantially altered by changing the selection of studies included, indicating that the pooled results were robust and insensitive to study selection. In contrast, altering the selection of studies included resulted in statistical changes to the pooled results, suggesting that the pooled results were more sensitive to study selection and less robust. All publication bias tests and sensitivity analyses of this study were performed using Stata 18.0 software.

## 3 Results

### 3.1 Literature search and eligible studies

**Figure 1:**
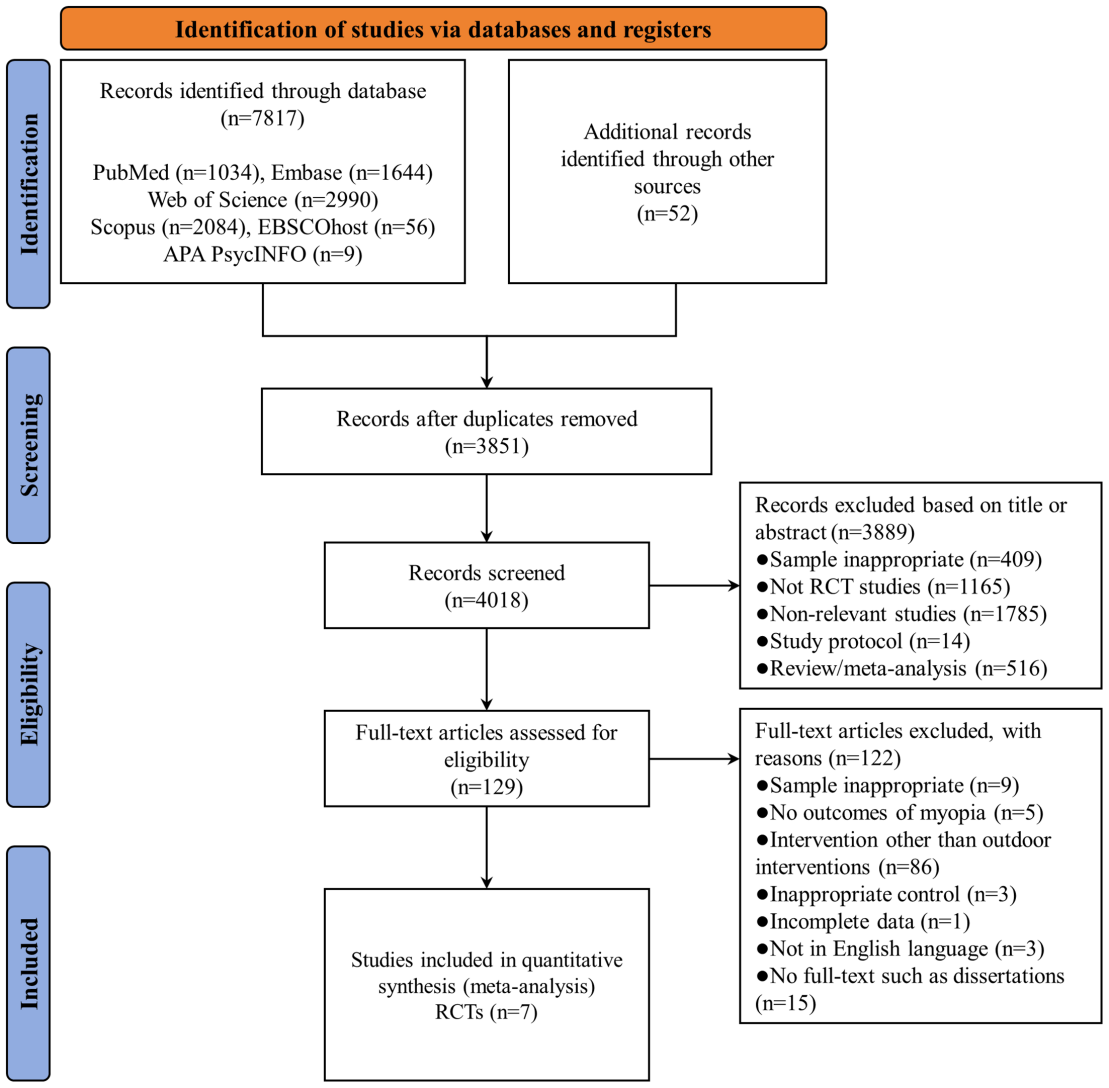
Flowchart of studies selection.

A total of 7,869 studies were identified through database searches, including PubMed (*n* = 1,034), Embase (*n* = 1,644), Web of Science (*n* = 2,990), Scopus (*n* = 2084), EBSCOhost (*n* = 56), APA PsycINFO (*n* = 9), and other sources (*n* = 52). After removing duplicate studies (*n* = 3,851), the titles and abstracts of 4,018 studies were screened for eligibility, and 3,889 references were eliminated due to samples inappropriate (*n* = 409), not RCT studies (*n* = 1,165), non-relevant studies (*n* = 1785), study protocol (*n* = 14), and review/meta-analysis (*n* = 516). Therefore, 129 studies were subjected to full-text review, 122 of which were deemed ineligible because the sample was inappropriate (*n* = 9), no outcomes of myopia (*n* = 5), intervention other than outdoor interventions (*n* = 86), inappropriate control (*n* = 3), incomplete date (*n* = 1), not in English language (*n* = 3), and no full-text such as dissertations (*n* = 15). Finally, 7 studies met the inclusion criteria and were included in the meta-analysis (39–45). A PRISMA flowchart of the literature search is presented in Figure 1.

### 3.2 Study characteristics

Seven full-text RCTs met inclusion criteria, all of which were conducted in China (39–45). The study populations consisted of students in Guangzhou (one study) (39), Shanghai (one study) (40), Shenyang (one study) (41), Wenzhou (one study) (42), Anyang (one study) (43), Yudu and Jiangxi (one study) (44), and Taiwan (one study) (45). In total, 4,778 subjects were assigned to the intervention group, with a mean age ranging from 6.61 to 10.09 years, while 4,659 were assigned to the control group, with a mean age ranging from 6.57 to 10.25 years. The length of the intervention ranged from 1 year to 3 years, the frequency from 5 to 7 times a week, and the duration from 40 to 60 min. The main characteristics of the seven RCTs are presented in Table 3.

**Table 3:** Main characteristics of included randomized controlled trials.

Included studies	Population	Age [Mean (SD)]	Total/M%	Intervention	Control	Outcome
He et al. (39) (China)	Children in grade 1 from 12 primary schools in Guangzhou	T: 6.61 (0.33) C: 6.57 (0.32)	T: 919/52.6% C: 929/54.6%	Time spent outdoors Length: 3 years Freq: 5 times a week Duration: 40 min	Treatment as usual	SER, AL, Myopia incidence
He et al. (40) (China)	Students aged 6–9 years from 24 primary schools in Shanghai	T: 7.30 (0.70) C: 7.20 (0.70)	T: 1878/52.9% C: 1608/52.8%	Time spent outdoors Length: 2 years Freq: 5 times a week Duration: 40 min	Treatment as usual	SER, AL, Myopia incidence
Jin et al. (41) (China)	Students of two primary and two junior high schools in Shenyang	T: 10.09 (2.35) C: 10.25 (2.33)	T: 214/54.2% C: 177/47.5%	Outdoor activities Length: 1 yearFreq: 5 times a week Duration: 40 min	No-intervention	SER, AL
Jingyi et al. (42) (China)	Students from three primary schools in Wenzhou	T: NR C: NR	T: 353/NR C: 366/NR	Outdoor activities Length: 1 yearFreq: 5 times a week Duration: 60 min	No-intervention	SER, AL
Li et al. (43) (China)	Students from 11 primary schools in Anyang	T: 8.38 (0.34) C: 8.35 (0.30)	T: 135/52.6% C: 133/57.1%	Time spent outdoors Length: 1 yearFreq: 7 times a week Duration: NR	Treatment as usual	SER, AL, Myopia incidence
Wang et al. (44) (China)	Children in grades 3 and 4 from 24 elementary school in Yudu and Jiangxi	T: 9.21 (0.62) C: 9.23 (0.62)	T: 1012/50.5% C: 1020/47.2%	Outdoor activities Length: 1 yearFreq: 5 times a week Duration: 120 min	No-intervention	SER, AL, Myopia incidence
Wu et al. (45) (China)	Grade 1 schoolchildren in 16 schools in Taiwan	T: NR C: NR	T: 267/55.1% C: 426/50.3%	Time spent outdoors Length: 1 yearFreq and Duration: 11 h weekly	Treatment as usual	SER, AL, Myopia incidence

T, test group; C, control group; M%, percentage of boys; SER, spherical equivalent refraction; AL, axial length.

### 3.3 Assessment of risk bias

**Figure 2:**
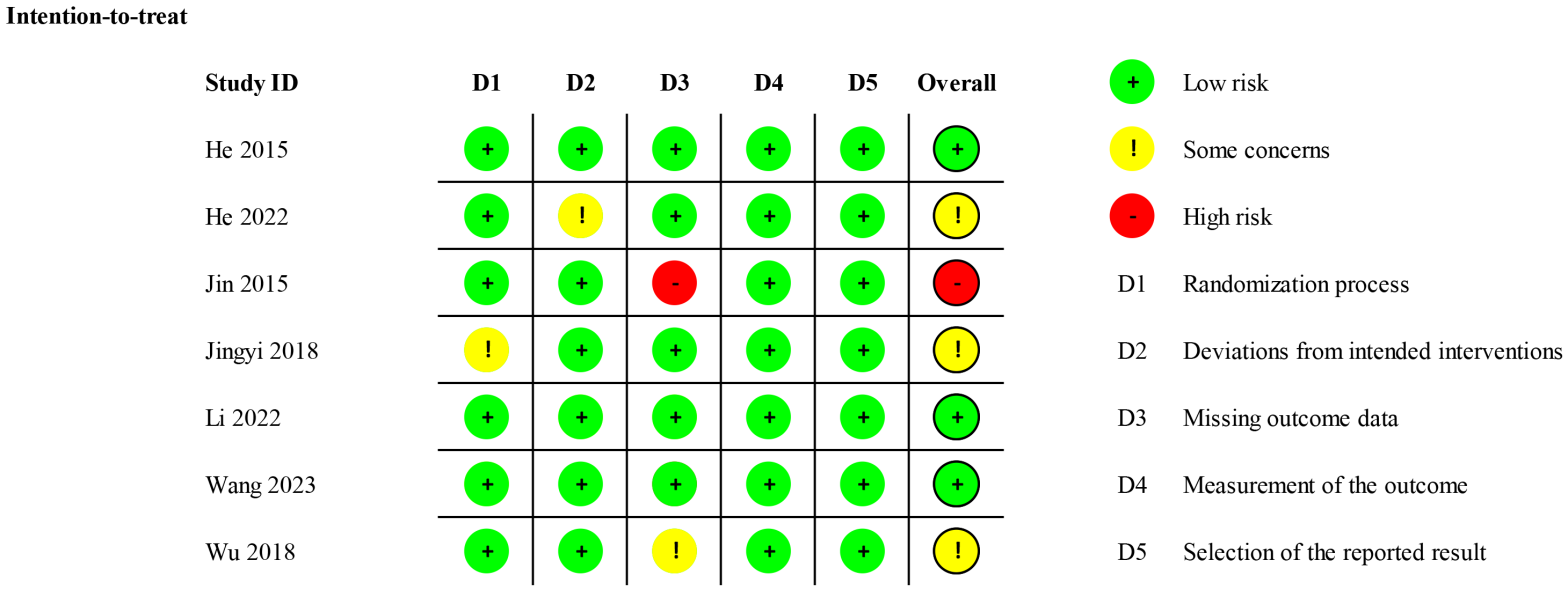
Risk of bias graph.

**Figure 3:**
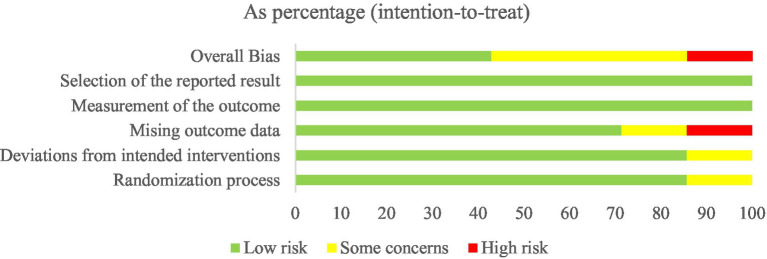
Risk of bias summary.

Six studies showed a low risk of bias in the randomization process (39–41, 43–45), and one study was assessed as having some concerns owing to the baseline differences (42). For deviations from intended interventions, six studies were considered low risk (39, 41–45), and one study was regarded as having some concerns because of the experimental context (40). For missing outcome data, five studies were considered low risk because the data for the outcome were available for all or nearly all randomized participants (39, 40, 42–44). One study had some concerns and one had high risk due to incomplete collection of participant data and lack of evidence that the result was not biased by missing outcome data (41, 45). The measurement outcome bias and selection of the reported result were low risk because all studies used appropriate methods to measure outcomes, and all measurements and data analyses were available in the results. The overall risk was low risk in three studies (39, 43, 44), some concerns in three studies (40, 42, 45), and high risk in one study (41). The Cochrane risk of bias assessment is presented in Figures 2, 3.

### 3.4 Meta-analysis

**Figure 4:**
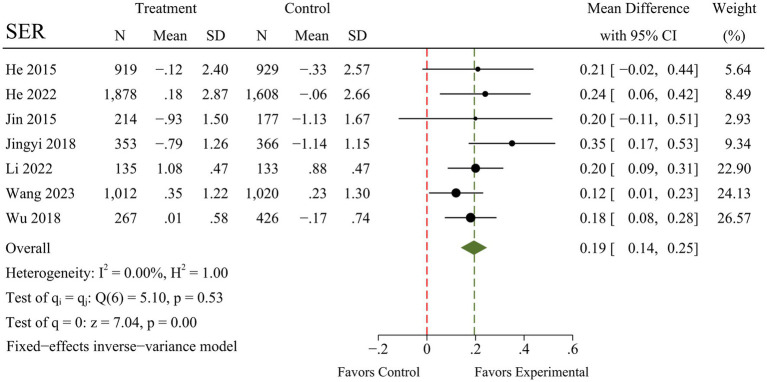
Forest plot of the effects of outdoor interventions on SER.

**Figure 5:**
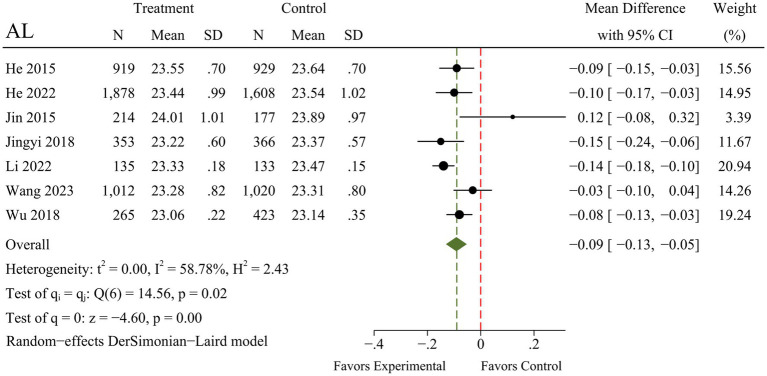
Forest plot of the effects of outdoor interventions on AL.

**Figure 6:**
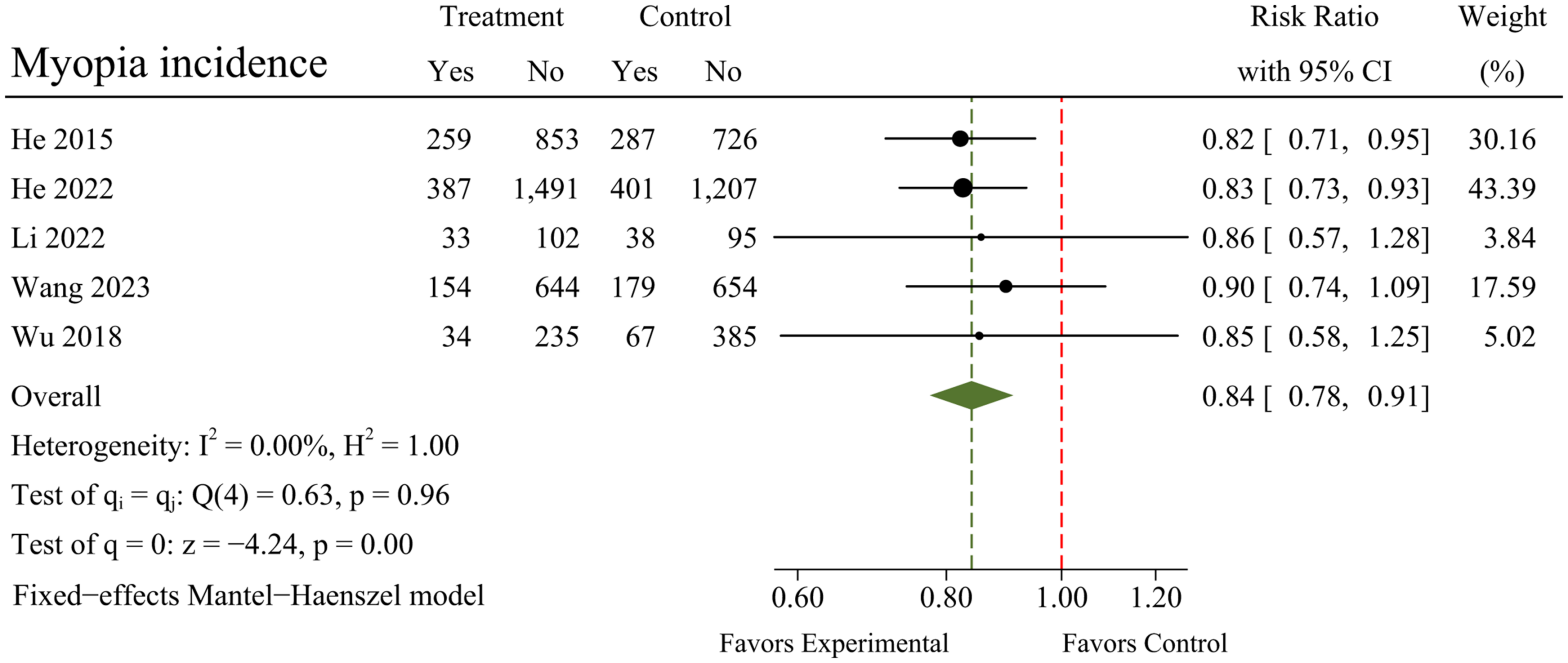
Forest plot of the effects of outdoor interventions on myopia incidence.

A total of seven RCTs were included in the meta-analysis, and heterogeneity was examined using the Chi-square test based on Q-test and *I*^2^ statistics, indicating that the pooled results for SER (*I*^2^ = 0.00%; *Q* = 5.10; *p* = 0.53) and myopia incidence (*I*^2^ = 0.00%; *Q* = 0.63; *p* = 0.96) showed no significant heterogeneity. The pooled results for AL, however, had moderate heterogeneity (*I*^2^ = 58.78%; *Q* = 14.56; *p* = 0.02). There were marked and statistically significant improvements in SER (MD = 0.19; 95% CI 0.14 to 0.25; *p* < 0.01), AL (MD = −0.09; 95% CI −0.13 to −0.05; *p* < 0.01), and myopia incidence (RR = 0.84; 95% CI 0.78 to 0.91; *p* < 0.01) following outdoor interventions. The results of the meta-analysis for each outcome are presented in Figures 4–6.

### 3.5 Sensitivity analysis

The results of the sensitivity analysis showed that the pooled results for SER, AL, and myopia incidence remained stable after excluding individual studies, indicating that these results were insensitive to study selection. The results of the sensitivity analysis for each outcome are presented in Table 4.

**Table 4:** Sensitivity analysis for outcomes by omitting individual studies.

Outcome	Study omitted	MD/RR	95% CI	
			Lower bound	Upper bound
SER	He et al. (39)	0.19	0.14	0.25
	He et al. (40)	0.19	0.13	0.24
	Jin et al. (41)	0.19	0.14	0.25
	Jingyi et al. (42)	0.18	0.12	0.23
	Li et al. (43)	0.19	0.13	0.25
	Wang et al. (44)	0.22	0.15	0.28
	Wu et al. (45)	0.20	0.14	0.26
AL	He et al. (39)	−0.09	−0.14	−0.04
	He et al. (40)	−0.09	−0.13	−0.04
	Jin et al. (41)	−0.10	−0.13	−0.07
	Jingyi et al. (42)	−0.08	−0.12	−0.04
	Li et al. (43)	−0.08	−0.12	−0.04
	Wang et al. (44)	−0.10	−0.14	−0.06
	Wu et al. (45)	−0.09	−0.14	−0.04
Myopia incidence	He et al. (39)	0.85	0.77	0.93
	He et al. (40)	0.85	0.76	0.95
	Li et al. (43)	0.84	0.77	0.91
	Wang et al. (44)	0.83	0.76	0.90
	Wu et al. (45)	0.84	0.77	0.91

MD, mean difference; RR, risk ratio; CI, confidence interval; SER, spherical equivalent refraction; AL, axial length.

### 3.6 Publication bias test

For SER and myopia incidence, the *p*-values for Egger’s test were 0.24 and 0.13, respectively, indicating that publication bias had no effect on this type of study. For AL, however, the *p*-value for Egger’s test was 0.01, indicating that there was publication bias in this result. The results of publication bias test for each outcome are presented in Table 5.

**Table 5:** Results of Egger’s test of each outcome.

Outcome	*t*	*p*-value	95% CI	
			Lower bound	Upper bound
SER	1.34	0.24	−0.10	0.32
AL	−4.02	0.01	−0.28	−0.06
Myopia incidence	−2.06	0.13	−0.53	0.11

CI, confidence interval; SER, spherical equivalent refraction; AL, axial length.

The trim and fill method was used to assess the stability of the pooled result for AL. The results showed that under both the fixed-effects model and random-effects model, the trim and fill adjustment using the linear method produced robust results. Specifically, under the fixed-effects model, the analysis estimated two missing studies after two iterations. After imputing these hypothetical studies, there was no statistically significant change in the pooled results. In the random-effects model, the analysis estimated one missing study after two iterations. After the hypothetical study was imputed, the pooled results remained unchanged and stable. The results of the trim and fill adjustment for AL are presented in Table 6.

**Table 6:** Results of trim and fill adjustment for AL.

Method	Studies	MD	95% CI	
			Lower bound	Upper bound
Fixed	Observed	−0.10	−0.12	−0.08
	Observed + Imputed	−0.11	−0.13	−0.10
Random	Observed	−0.09	−0.13	−0.05
	Observed + Imputed	−0.10	−0.14	−0.06

MD, mean difference; CI, confidence interval.

## 4 Discussion

The objective of this systematic review and meta-analysis was to evaluate the overall efficacy of outdoor interventions for myopia in children and adolescents, and to provide evidence for the prevention and control of myopia. The pooled results of the meta-analysis demonstrated that outdoor interventions effectively improved SER (MD = 0.19; 95% CI 0.14 to 0.25; *p* < 0.01), AL (MD = −0.09; 95% CI −0.13 to −0.05; *p* < 0.01), and myopia incidence (RR = 0.84; 95% CI 0.78 to 0.91; *p* < 0.01), indicating that outdoor interventions had a beneficial effect on the prevention and control of myopia in children and adolescents. Although there was a moderate heterogeneity and publication bias in some of the pooled results, these results did not change statistically after adjustment using the stepwise elimination and trim and fill methods, implying that the evidence provided by this study was reliable.

Overall, outdoor interventions appear to be a promising approach to preventing and controlling myopia in children and adolescents, and the mechanisms may be explained from several perspectives. Dopamine, a neurotransmitter closely linked to ocular development, has been demonstrated to be beneficial in inhibiting the increase of AL (46, 47), and the protective effect of outdoor interventions on myopia in children and adolescents may be mediated through light stimulation of retinal dopamine production and release (48). When children and adolescents are in an outdoor environment, retinal dopaminergic pathways can be activated through the influence of outdoor light and dopamine availability increased (49–51), with the increase of AL being suppressed (52, 53). A RCT examining the relationship between outdoor light intensity and myopia showed that children and adolescents exposed to light intensity of 1,000 lux or higher experienced significant improvements in both SER and AL, in addition to a reduction in myopia incidence compared with the control group (45). Furthermore, a non-negligible explanation for the impact of being outdoors on myopic progression may be the profound differences in the pattern of retinal defocus generated indoors and outdoors (54). Contrasting indoor scenes with outdoors reveals a marked increase in the level of hyperopic defocus for both near and distant fixation while indoors, and this persistent hyperopic defocus contributes to the progression of myopia. In contrast, being outdoors may be protective on the basis that it provides minimal amounts of peripheral defocus and hence may provide a so-called STOP signal for the development of myopia (54).

The pooled results in this study showed that outdoor interventions, including engaging in outdoor activities and increasing time spent outdoors, were effective in preventing and controlling the progression of myopia in children and adolescents compared with the control group, which is promising to resolve the current disagreement in research in this area. The reason for this divergence may stem from the differences in study populations, geographical backgrounds, interventions and outcomes, as well as the definitional criteria employed. The RCTs included in this study were all conducted within China, a country with a high myopia incidence, suggesting that outdoor interventions may exert more substantial preventive and control effects in specific populations. However, this evidence should be extended to a wider range of populations and other countries with lower rates of myopia, and examined for specific applications to comprehensively assess the effectiveness of outdoor interventions for the prevention and control of myopia in different contexts, so as to develop differentiated intervention programs according to the characteristics of different populations and regions.

In addition, although several RCTs have restricted the duration of interventions to 40–60 min, the optimal time for outdoor interventions remains unclear. This ambiguity implies that the “dose–response” relationship between outdoor intervention and myopia-related outcomes is not well understood. Similarly, when should outdoor interventions be implemented (midday versus before and after school)? What is the ideal age for myopia prevention and control? And will myopia rebound after cessation of outdoor interventions? These questions necessitate further investigation in future studies to compare the effectiveness of different intervention modes in the prevention and control of myopia, thereby optimizing the specific implementation strategies of outdoor interventions. Finally, considering that sunlight exposure may serve as a risk factor for certain diseases, including skin cancer or pterygium, it is essential to take environmental conditions (such as light intensity and climate temperature) into account during the implementation of interventions (55, 56). Appropriate preventive measures should be adopted to ensure the safety of interventions, thereby minimizing potential health risks.

In summary, the findings of this study suggest that outdoor interventions effectively contributed to the prevention and control of myopia in children and adolescents, positively impacting SER, AL, and myopia incidence. Children and adolescents are at a critical stage in healthy physical and mental development, and the cumulative effect caused by heavy educational burden and information overflow may increase the risk of myopia in children and adolescents. Outdoor interventions were characterized by low risk and high therapeutic benefits and could serve as alternative or adjuvant approaches to medication for the treatment of myopia. Therefore, considering the advantages in terms of safety and efficacy, outdoor interventions may be considered as a preferred intervention for the treatment of myopia in children and adolescents, while susceptibility to diseases associated with sunlight, particularly UV exposure, must be taken into account. Appropriate medication can be adopted in accordance with specific conditions to further enhance the therapeutic effect, and the improvement in myopia and related indicators can be maximized through this comprehensive treatment in children and adolescents.

### 4.1 Limitations

The present systematic review and meta-analysis had several limitations. First of all, due to the limited number of RCTs that met the inclusion criteria, sources of heterogeneity between studies may not be explored and discussed. Moreover, this study investigated the overall efficacy of outdoor interventions for myopia in children and adolescents as a whole, and was not divided into myopic and non-myopic children and adolescents to be analyzed separately on this basis. Finally, since the included studies were conducted within China, the generality of the results to other populations and regions needs to be further examined, in order to comprehensively evaluate the efficacy of outdoor intervention on the prevention and control of myopia in different backgrounds.

## 5 Conclusion

The objective of this systematic review and meta-analysis was to evaluate the overall efficacy of outdoor interventions for myopia in children and adolescents, and to provide evidence for the prevention and control of myopia. Outdoor interventions effectively contributed to the prevention and control of myopia in children and adolescents, positively impacting SER, AL, and myopia incidence. Moreover, outdoor interventions were characterized by low risk and high therapeutic benefits and could serve as alternative or adjuvant approaches to medication for the treatment of myopia. Therefore, considering the advantages in terms of safety and efficacy, outdoor interventions may be considered as a preferred intervention for the treatment of myopia in children and adolescents, while susceptibility to diseases associated with sunlight, particularly UV exposure, must be taken into account.

## Data availability statement

The original contributions presented in the study are included in the article/supplementary material, further inquiries can be directed to the corresponding author.

## Author contributions

ZYM: Conceptualization, Data curation, Methodology, Resources, Software, Visualization, Writing – original draft, Writing – review & editing. YZZ: Formal analysis, Software, Validation, Writing – original draft. WFJ: Conceptualization, Formal analysis, Software, Writing – original draft. CFL: Methodology, Resources, Visualization, Writing – original draft. SLL: Data curation, Methodology, Software, Visualization, Writing – original draft. CYC: Conceptualization, Data curation, Resources, Writing – review & editing. SL: Funding acquisition, Methodology, Project administration, Supervision, Writing – review & editing.
